# Dr. Henry J. Fenn

**Published:** 1916-05

**Authors:** 


**Dr. Henry J. Fenn,** a dentist of Albion, died April 11 of apoplexy, aged 47.

